# 儿童及青少年血清中多环芳烃浓度水平及健康风险评估

**DOI:** 10.3724/SP.J.1123.2025.07015

**Published:** 2025-10-08

**Authors:** Lipan WANG, Zhiyong DENG, Shiyu SHI, Huawei WANG, Kang’an LIU, Xiang LI, Mingye ZHANG, Surong MEI

**Affiliations:** 1.华中科技大学公共卫生学院环境医学研究所，教育部环境与健康重点实验室，湖北 武汉 430030; 1. Institute of Environmental Medicine，School of Public Health，Huazhong University of Science and Technology，Key Laboratory of Environment & Health of Ministry of Education，Wuhan 430030，China; 2.武汉市洪山区疾病预防控制中心，湖北 武汉 430070; 2. Hongshan District Center for Disease Control and Prevention，Wuhan 430070，China

**Keywords:** 气相色谱-串联质谱, 多环芳烃, 血清, 儿童, 青少年, 暴露特征, 健康风险评估, gas chromatography-tandem mass spectrometry （GC-MS/MS）, polycyclic aromatic hydrocarbon （PAHs）, serum, children, adolescents, exposure profile, health risk assessment

## Abstract

多环芳烃（PAHs）是一类具有潜在致癌性的持久性有机污染物，广泛存在于空气、土壤和水体等环境介质中。儿童及青少年由于生理发育尚未成熟，对PAHs更为敏感，其体内PAHs的暴露状况及相关健康风险亟需深入研究。本研究以我国西部地区某市1 096名6~18岁儿童及青少年为研究对象，采用固相萃取结合气相色谱-串联质谱技术（SPE-GC-MS/MS）同时测定血清中12种PAHs的浓度，并运用多元线性回归模型探究人口学特征、生活方式、社会经济因素和饮食习惯对血清中PAHs浓度的影响。根据血清中PAHs的浓度估算其每日总摄入量（TEDI），结合参考剂量（RfD）计算风险熵（HQ），以评估个体的非致癌健康风险；同时，通过计算苯并［*a*］芘当量（BaPeq）浓度进一步评估其致癌风险。结果表明，6种PAHs的检出率大于50%，依次为蒽（87.2%）、菲（76.3%）、芴（69.3%）、苊（62.1%）、芘（54.3%）和苊烯（53.7%），其中含量最高的是菲，中位数质量浓度为3.03 ng/mL。年龄与这6种PAHs浓度均呈正相关；超重及肥胖者的苊烯和苊浓度较低；母亲教育水平越高，子女血清PAHs浓度越低；蔬菜摄入可增大苊浓度，而牛奶摄入可减小苊、苊烯和菲浓度；饮用桶装水与蒽和菲浓度升高相关。风险评估显示，芘的HQ均小于1，提示芘的非致癌风险较低；蒽和菲贡献了约85%的总BaPeq浓度，是主要的致癌风险单体。综上所述，该地区儿童及青少年普遍暴露于PAHs，年龄、体重质量指数（BMI）、母亲教育水平和饮食可能是影响血清中PAHs浓度的主要因素。本研究所纳入人群非致癌风险总体较低，蒽和菲是最具致癌潜力的单体，未来应加强对儿童及青少年PAHs长期低剂量暴露的监测与健康干预。

## Abstract (en)

Polycyclic aromatic hydrocarbons （PAHs） are a class of persistent organic pollutants that are widely present in various environmental media such as air， soil， and water. They are highly environmentally stable and are mainly derived from combustion processes and industrial emissions. Although numerous studies have shown that PAHs are carcinogenic， mutagenic， and neurotoxic， their exposure levels and associated health risks in specific populations require further study. Children and adolescents are in a critical stage of growth and development， with immature physiological and immune systems， making them more sensitive to PAHs than adults. Hence， their internal exposure levels to PAHs and the associated health risks warrant particular attention. However， most of the existing studies have focused on adults， and there are relatively few studies on PAHs exposure in children and adolescents. Therefore， a total of 1 096 children and adolescents aged 6-18 years in a city located in western China were investigated. The concentrations of 12 PAHs in serum samples were simultaneously determined by solid-phase extraction coupled with gas chromatography-tandem mass spectrometry （SPE-GC-MS/MS）， and the effects of demographic characteristics， lifestyles， socioeconomic factors， and dietary habits on the concentrations of PAHs in serum were investigated using multiple linear regression models. The total estimated daily intake （TEDI） of PAHs was calculated based on their serum concentrations， and the hazard quotient （HQ） was derived using the reference dose （RfD） to evaluate non-carcinogenic health risk. Meanwhile， carcinogenic risk was evaluated based on the benzo［*a*］pyrene equivalent （BaPeq） concentrations. The results showed that six PAHs were detected in the serum of more than 50% of children and adolescents， in descending order， with anthracene （Ant） （87.2%）， phenanthrene （Phe） （76.3%）， fluorene （Flu） （69.3%）， acenaphthene （Acp） （62.1%）， pyrene （Pyr） （54.3%）， and acenaphthylene （AcPy） （53.7%）. Phe showed the highest median mass concentration of 3.03 ng/mL. Age was positively associated with serum concentrations of all six PAHs （AcPy： *β*=0.097， 95% CI： 0.033–0.160； Acp： *β*=0.103， 95% CI： 0.032–0.174； Ant： *β*=0.056， 95% CI： 0.016–0.097； Flu： *β*=0.085， 95% CI： 0.009–0.162； Phe： *β*=0.098， 95% CI： 0.029–0.167； Pyr： *β*=0.136， 95% CI： 0.078–0.195）； overweight and obese participants exhibited lower levels of AcPy （*β*=–0.538， 95% CI： –1.022– –0.053） and Acp （*β*=–0.566， 95% CI： –1.104– –0.028）； lower PAHs levels were also found in children and adolescents from families with higher maternal education； higher frequency of vegetable intake was positively associated with Acp （*β*=0.088， 95% CI： 0.012–0.165）， while higher milk intake was inversely associated with AcPy （*β*=–0.072， 95% CI： –0.145– –0.000）， Acp （*β*=–0.087， 95% CI： –0.167– –0.007） and Phe （*β*=–0.095， 95% CI： –0.173– –0.017）； the use of water cooler jug was positively linked to serum levels of Ant （*β*=0.462， 95% CI： 0.149–0.774） and Phe （*β*=0.898， 95% CI： 0.367–1.428）. HQ values for Pyr were all less than 1， indicating that the level of Pyr exposure in the serum is insufficient to pose a significant non-carcinogenic health risk. The carcinogenic risk assessment revealed that Ant and Phe were the major individual compounds contributing to the carcinogenic risk posed by PAHs exposure in children and adolescents， accounting for 85% of the total BaPeq concentration. In summary， exposure to PAHs is common among children and adolescents in this region. Age， body mass index （BMI）， maternal education level， and diet may be the main factors influencing serum PAHs concentrations. The overall non-carcinogenic risk among the studied population was low， and Ant and Phe were identified as the individual compounds with the greatest carcinogenic potential. These findings highlight the need for strengthened monitoring and targeted health interventions regarding long-term， low-dose PAHs exposure in children and adolescents.

多环芳烃（PAHs）是一类由两个或以上苯环构成的持久性有机污染物，广泛存在于空气、土壤和水体等环境介质中^［1-3］^。按照苯环数目，PAHs通常被划分为低相对分子质量（2~3环）与高相对分子质量（≥4环）两类。PAHs一般在燃烧和工业排放过程中产生^［4，5］^，可通过空气吸入、食物摄入及皮肤接触等多种途径进入人体，蓄积在组织和器官中，进而对人体健康构成潜在威胁。流行病学和毒理学研究表明，PAHs长期暴露可能产生致癌、致突变和神经毒性作用^［6-8］^。美国环保局（USEPA）自1979年起将16种PAHs列为优先控制的污染物^［9］^。近年来，随着工业化和城市化进程的加快，PAHs排放呈持续增长趋势。已有研究表明，湖泊沉积物中PAHs浓度自20世纪80年代以来显著上升，这一趋势与我国城市化和工业化的快速发展高度一致，提示人类活动对环境中PAHs累积具有重要影响^［10］^。

虽然已有研究明确了PAHs的多种毒性效应，但其在人群中的暴露情况及健康影响仍存在诸多不确定性。相较于传统的外暴露评估方法，内暴露监测能够综合反映个体在一定时期内通过多种途径摄入PAHs的总体暴露负荷，从而提供更真实、全面的暴露信息。人体生物监测（HBM）是评估环境污染物内暴露水平的有效手段^［11］^。目前，PAHs的暴露评估多采用尿液中代谢产物（如OH-PAHs）作为生物标志物，适用于反映短期或近期暴露^［12］^。然而，血清中PAHs母体化合物的检测可更直接反映其在体内的实际累积情况，尤其对评估脂溶性PAHs的长期暴露负荷具有重要价值^［13］^。因此，采用血清样本进行PAHs监测有助于从一个新的视角揭示其暴露特征，更具参考意义。

儿童及青少年正处于生长发育的关键时期，其生理和免疫系统尚未完全成熟，因此对环境污染物的敏感性较成人更高^［14］^。研究表明，PAHs的长期暴露可能导致儿童及青少年出现内分泌紊乱、神经发育障碍及生长发育迟缓等健康问题^［15］^，且其致癌风险相较于成年人更高^［16］^。考虑到这一人群的特殊性，评估儿童及青少年群体的PAHs内暴露水平和健康风险显得尤为重要。然而，目前关于儿童及青少年群体暴露于PAHs的研究相对较少，且大多数研究集中于成人，存在明显的研究空白。因此，开展针对儿童及青少年群体的PAHs内暴露水平监测及健康风险评估，对于全面揭示其健康危害具有重要意义。

PAHs的内暴露水平受多种因素的影响，包括人口学特征、社会经济状况、生活方式和饮食习惯等。不同的环境因素和个人行为可能导致个体暴露于不同浓度的PAHs^［17，18］^。因此，厘清这些因素与血清中PAHs浓度之间的关系，对于制定更有针对性的暴露干预措施具有重要意义。本研究选取我国西部地区某市儿童及青少年作为研究对象，采用固相萃取结合气相色谱-串联质谱技术（SPE-GC-MS/MS）测定血清中12种PAHs的内暴露水平，阐明与暴露水平相关的影响因素，并评估PAHs的致癌和非致癌风险。

## 1 实验部分

### 1.1 仪器、试剂与材料

8890气相色谱仪-7010B三重四极杆质谱联用系统及真空固相萃取装置（美国Agilent科技有限公司），DOA-P504-BN隔膜真空泵（美国GAST公司），CT-1氮气发生器（武汉科林普丰仪器有限公司），EFAA-DC24-RT氮吹仪（上海安谱实验科技股份有限公司），MX-S涡旋振荡仪（美国Scilogex公司）。

Oasis^®^ PRiME HLB固相萃取柱（1 mL/30 mg，美国Waters公司），色谱纯二氯甲烷、丙酮（美国Fisher公司）；色谱纯甲醇、正己烷（德国Merck公司），分析纯88%甲酸（国药集团有限公司），纯净水（杭州娃哈哈集团有限公司）。

PAHs标准品：12种PAHs混合标准溶液（100 µg/mL，货号：1ST4360-100H）购于天津阿尔塔科技有限公司。具体名单详见附表1（www.chrom-China.com）。

同位素内标：芘-D_10_同位素内标标准溶液（100 μg/mL，加拿大Toronto Research Chemicals公司）。

### 1.2 标准溶液配制

将PAHs标准品溶于丙酮，配制成质量浓度为20 μg/mL的12种PAHs混合标准贮备液。将混合标准贮备液用丙酮稀释得到质量浓度为2 μg/mL的混合标准应用溶液，并用丙酮配制成质量浓度为0.2、0.5、1、2、5、10、20、50、100、200、500、1 000 ng/mL的系列标准溶液。同位素内标芘-D_10_溶液配制成质量浓度为20 ng/mL的内标贮备液。上述所有标准溶液均在-20 ℃的冰箱中保存备用。

### 1.3 血液样品采集

本研究选取了我国西部地区某市的1 388名6~18岁儿童及青少年作为研究对象，采用问卷调查收集了研究对象的性别、年龄、身高、体重、体质指数（BMI）、体育锻炼和膳食资料等信息。采集研究对象静脉血后离心取血清，-80 ℃保存备用。将血清样本不足或缺失以及膳食调查数据缺失的参与者剔除后，最终1 096名儿童及青少年纳入后续分析。本研究得到柳州市妇幼保健院伦理委员会批准，所有参与者及其监护人均签署知情同意书。

### 1.4 样品前处理

取200 μL血清样品于离心管中，加入10 μL质量浓度为20 ng/mL的芘-D_10_内标溶液，涡旋混匀，置于4 ℃冰箱中静置过夜。向样品中加入200 μL 15%甲酸水溶液并混匀；使用Oasis^®^ PRiME HLB固相萃取柱净化（预先用3 mL二氯甲烷、3 mL甲醇和3 mL纯净水活化小柱），样品上柱后用1 mL甲醇-水（1∶6，v/v）溶液淋洗，真空抽干，再依次用3 mL二氯甲烷和3 mL正己烷进行洗脱；收集全部洗脱液并用氮气浓缩至近干，复溶于100 μL丙酮溶液，转入样品瓶待测。

### 1.5 分析条件

色谱条件 Agilent DB-5MS毛细管色谱柱（30 m×0.25 mm×0.25 μm）；高纯氦气作为载气，流速1.2 mL/min；进样口温度270 ℃；不分流进样，进样量1 μL。色谱柱升温程序：初始温度70 ℃，保持2 min，以25 ℃/min升温至150 ℃；以3 ℃/min升至200 ℃，保持2 min；以8 ℃/min升至300 ℃，保持8 min。

质谱条件 电子轰击离子源（EI），电离电压70 eV；离子源温度300 ℃，传输线温度300 ℃；溶剂延迟时间6 min；扫描模式为多反应监测（MRM），内标法定量。更详细的方法学描述参考课题组前期研究工作^［19］^。质谱参数及定量分析相关数据详见附表2和附表3）。

### 1.6 脂质标准化浓度和BMI Z值的计算

血清中总胆固醇（total cholesterol，TC）和甘油三酯（triglycerides，TG）含量使用全自动生化分析仪（Cobas 8000，罗氏公司）进行测定，污染物的脂质标准化浓度（ng/g脂质）由式（1）和式（2） ^［20］^进行计算。


Total lipids (g/L)=1.12×TC(g/L)+1.33×TG(g/L)+1.48(g/L) (1)

$$C_{lipid⁃standardized}\;(ng/g\;lipid)=\frac{C_{wet⁃weighted}\;(ng/mL)\times 1000\;}{Total\;lipids\;(g/L)}$$ (2)

其中Total lipids为血清总脂质，*C*_wet-weighted_为PAH单体的检测浓度，*C*_lipid-standardized_为PAH单体的脂质标准化浓度。

为了确保不同性别和年龄间的数据可比性，本研究采用了世界卫生组织（World Health Organization，WHO）制定的5~19岁儿童及青少年生长发育参考标准，并使用LMS（Lambda-Mu-Sigma）法计算BMI Z值（BMI Z-score）。BMI Z值定义为个体BMI相对于同年龄、同性别人群中位数的标准差分数，反映个体在参考人群中的相对位置。根据WHO建议，BMI Z值大于1个标准差（SD）判定为超重，BMI Z值大于2SD判定为肥胖^［21］^。LMS法的计算公式见式（3）。


$$BMI\;Z_{score}=\frac{{\left(\frac{BMI}{M}\right)}^{L}-1\;}{L\times S\;}$$ (3)

其中BMI（kg/m^2^）为体质指数；*L*是偏态系数，用于调整BMI分布的偏度；*M*是特定性别和年龄下的BMI中位数；*S*是特定性别和年龄下的BMI的标准差乘以变异系数。

### 1.7 统计分析

本研究对检出率大于50%的PAHs进行统计分析，低于定量限（LOQ）的浓度以LOQ/ $\sqrt[{}]{2}$ 计算。由于污染物浓度呈偏态分布，故对其进行自然对数转换后，纳入多元线性回归模型探究影响血清中PAHs浓度水平的关键因素。采用R4.4.3进行统计分析。双侧检验水准*α*=0.05。

### 1.8 健康风险评估

#### 1.8.1 非致癌健康风险评估

在血清浓度达到稳态的假设前提下（即体内吸收与排出速率相等，血清浓度不随时间显著变化），可采用药代动力学模型反推出个体PAHs每日总摄入量（total estimated daily intake， TEDI）。随后，采用风险熵（hazard quotient， HQ）评估PAHs的非致癌健康风险。当HQ<1时，表示非致癌健康风险可接受，HQ>1时，则提示可能存在非致癌健康风险^［22］^。计算公式如下：


$$v\frac{dC}{dt}=TEDI\times BW\times A\times f-b\times V\times C\;$$ (4)

$$HQ=\frac{TEDI}{RfD}$$ (5)

其中，*C*（ng/mL）为血清中单个PAHs的质量浓度；*V*（mL）为血液总量（男性：按体重的8%计算，即80 mL/kg；女性：7%，即70 mL/kg）；BW是体重（kg）；A为吸收速率（无量纲）；*f*为吸收剂量分布在血液中的比例（无量纲）；b为PAHs在体内的消除速率常数；RfD为PAHs的每日口服参考剂量（μg/（kg bw·day）），表示在终身每日暴露的前提下，被认为不会引起可识别不良健康效应的剂量水平。

#### 1.8.2 致癌健康风险评估

考虑到多种PAHs已被国际癌症研究机构（IARC）列为明确或潜在的人类致癌物，本研究进一步采用苯并［*a*］芘当量（BaPeq）浓度评估其致癌风险。各PAH单体的BaPeq浓度可按以下公式计算：


$$BaPeq=TEF\times C_{serum}$$ (6)

其中*C*_serum_是血清中PAH单体的浓度；TEF为其毒性当量因子，表示该PAH单体相对于苯并［*a*］芘的致癌能力。本研究采用Nisbet和LaGoy提出的毒性当量因子（TEF）作为参考依据^［23］^。对所有具备TEF值的PAH单体计算其BaPeq浓度并求和，即可估算PAHs的总体致癌效力。

## 2 结果与讨论

### 2.1 人口学特征

1 096名参与者的平均年龄为（12.31±3.23）岁，BMI Z值为1.17±0.04，其中男孩506人（46.17%），肥胖或超重225人（20.53%）。母亲受教育水平至少为大学学历的有278人（25.36%），194人的家庭月收入超过8 000元（17.70%），860人报告无二手烟暴露史（78.47%），316人（28.83%）很少进行规律性的体育锻炼，且62.63%的参与者都是饮用自来水。

### 2.2 血清中PAHs的浓度水平

本研究对血清中12种PAHs进行了检测，其中6种PAH的检出率超过50%。从高到低依次为蒽（87.2%）、菲（76.3%）、芴（69.3%）、苊（62.1%）、芘（54.3%）和苊烯（53.7%），中位质量浓度依次为0.54、3.03、1.06、0.32、0.03、0.04 ng/mL（见表1）。值得注意的是，虽然蒽的检出率最高，但菲的中位质量浓度显著高于其他PAHs，几乎是蒽的6倍。这种检出频率与浓度水平的分离现象，提示不同PAHs可能存在差异化的暴露途径或毒代动力学行为^［24］^。低相对分子质量PAHs（蒽、菲、芴、苊、苊烯）在检出率和浓度上均占主导地位，这与Yin等^［25］^在脐带血清中的观察结果相似。这表明不同人群暴露的PAHs均以低相对分子质量组分为主，可能反映了此类污染物在环境介质中相似的释放来源。低相对分子质量PAHs主要来源于石油泄漏、化石燃料和生物质的不完全燃烧，在城市环境中其主要来源是交通燃油排放和燃煤^［26］^。相较于高相对分子质量PAHs，低相对分子质量PAHs的亨利常数较高，挥发性更强，更容易通过呼吸或摄入等途径进入人体，从而增加其被吸收的可能性^［27］^。对于高相对分子质量PAHs（如苯并［*a*］芘、苯并［*b*］荧蒽、苯并［*k*］荧蒽），尽管其检出率较低且浓度多在定量限（LOQ）以下，但由于其强致癌性，即使是低浓度仍值得关注，提示需持续监测和评估其健康风险。此外，与沙特^［28］^、印度^［29］^等其他发展中国家儿童相比，本研究中我国西部地区儿童及青少年血清中PAHs浓度总体处于较低水平。例如，印度儿童血清中菲和蒽的中位数质量浓度分别为9.0和3.6 ng/mL，沙特儿童血清中相应质量浓度分别为9.3和239.5 ng/mL，而本研究中分别为3.03和0.54 ng/mL，这种差异可能与不同国家在环境污染治理、交通能源结构、饮食模式及生活方式等方面的差异密切相关。

**表1:** 儿童及青少年血清中多环芳烃含量分布

Compound	Detection rate/%	*C*_wet-weighted_/（ng/mL）					*C*_lipid-standardized_/（ng/g lipid）			
		Geometric mean	Percentile				Geometric mean	Percentile		
			P25	P50	P75			P25	P50	P75
Ant	87.2	0.33	0.19	0.54	1.23		75.24	41.94	122.30	277.93
Phe	76.3	1.11	0.11	3.03	14.44		249.82	26.58	690.68	3261.51
Flu	69.3	0.20	<LOQ	1.06	4.41		44.37	<LOQ	240.44	1004.89
Acp	62.1	0.06	<LOQ	0.32	1.24		13.86	<LOQ	68.84	283.21
Pyr	54.3	0.06	<LOQ	0.03	0.32		3.69	<LOQ	7.70	73.37
AcPy	53.7	0.02	<LOQ	0.04	0.41		4.51	<LOQ	8.78	99.51
Flt	42.0	0.03	<LOQ	<LOQ	0.34		5.97	<LOQ	<LOQ	78.78
Chr	30.7	0.01	<LOQ	<LOQ	0.03		2.11	<LOQ	<LOQ	6.74
BaA	30.4	0.01	<LOQ	<LOQ	0.02		2.73	<LOQ	<LOQ	3.81
BaP	22.7	0.01	<LOQ	<LOQ	<LOQ		1.36	<LOQ	<LOQ	<LOQ
BbF	18.9	0.01	<LOQ	<LOQ	<LOQ		1.30	<LOQ	<LOQ	<LOQ
BkF	17.5	0.01	<LOQ	<LOQ	<LOQ		1.41	<LOQ	<LOQ	<LOQ

Ant： anthracene； Phe： phenanthrene； Flu： fluorene； Acp： acenaphthene； Pyr： pyrene； AcPy： acenaphthylene； Flt： fluoranthene； Chr： chrysene； BaA： benz［*a*］anthracene； BaP： benzo［*a*］pyrene； BbF： benzo［*b*］fluoranthene； BkF： benzo［*k*］fluoranthene. LOQ： limit of quantification.

### 2.3 血清中PAHs浓度的影响因素分析

将血清化学污染物浓度作为因变量，人口学资料、生活习惯、家庭情况和膳食资料作为自变量建立多元线性回归方程，分析影响血清中PAHs浓度的因素（见表2）。结果显示，年龄、BMI、母亲教育水平、膳食因素（蔬菜、牛奶）和桶装水的饮用对血清中PAHs的浓度产生了显著影响。年龄与血清中多个PAHs的浓度呈正相关（苊烯：*β*=0.097，95% CI：0.033~0.160；苊：*β*=0.103，95% CI：0.032~ 0.174；蒽：*β*=0.056，95% CI：0.016~0.097；芴：*β*=0.085，95% CI：0.009~0.162；菲：*β*=0.098，95% CI：0.029~0.167；芘：*β*=0.136，95% CI：0.078~0.195）。随着年龄增长，PAHs在体内逐渐蓄积，可能与其作为持久性有机污染物所具有的较长半衰期有关。然而，本研究发现超重和肥胖儿童及青少年血清中苊烯和苊的浓度较低（苊烯：*β*=-0.538，95% CI：-1.022~-0.053；苊：*β*=-0.566，95% CI：-1.104~-0.028），这一现象可能受脂质分布和代谢活性双重因素影响。Moriceau等^［30］^的研究表明，BMI与持久性有机污染物在脂肪组织与血清间的分布比值呈显著正相关，提示BMI较高个体体内的PAHs更倾向于储存在脂肪组织中，导致单位体积血清中PAHs浓度相对降低。同时，肥胖相关的炎症状态可能激活脂肪组织中的某些代谢酶，如细胞色素P450 1A1（CYP1A1），从而加速PAHs的转化与清除^［31］^。

与You等^［18］^对中国城市居民的研究结论一致，本研究中母亲教育程度较高的家庭，儿童及青少年血清中PAHs浓度普遍较低。这可能与高教育水平家庭通常拥有更优越的居住环境、更健康的饮食结构以及更强的污染防护意识等因素有关。Govarts等^［32］^基于欧洲多国的大规模人体生物监测数据同样表明，儿童和青少年体内PAHs暴露水平与其家庭教育程度呈显著负相关。蔬菜摄入频率与血清苊浓度呈正相关（*β*=0.088，95%CI：0.012~0.165），提示部分蔬菜可能在生长或烹饪过程中引入PAHs^［33，34］^。相反，牛奶摄入与苊、苊烯及菲等浓度呈负相关，这可能归因于乳脂对PAHs的竞争性结合或钙离子促进肠道螯合排泄，尽管目前在人群中相关研究仍有限。桶装水的饮用与血清中蒽和菲的浓度呈正相关，这可能是因为塑料容器（尤其是聚对苯二甲酸乙二醇酯，PET）可释放包括蒽、萘、菲等低相对分子质量PAHs，且随着贮存时间的延长，水中的PAHs浓度会不断上升^［35］^，提示饮用水源可能是体内PAHs的一个潜在暴露路径。此外，一些市售桶装水容器使用回收塑料作为原料，其添加剂与降解副产物亦可能增加PAHs的暴露。

本研究主要依赖自报膳食和生活习惯数据，可能存在回忆偏差或报告不准确的问题。未来的研究可以通过更精确的饮食记录和长期暴露监测来获得更加准确的暴露数据。此外，尽管本研究探讨了多种因素对血清中PAHs浓度的影响，但其他潜在因素如遗传因素、环境污染的长期暴露历史等未能纳入分析。未来研究可以进一步探索这些因素对PAHs浓度的潜在影响。

**表2:** 血清中多环芳烃含量影响因素的多元线性回归分析结果

Influencing factor		Type	*β* （95% CI）					
			AcPy	Acp	Ant	Flu	Phe	Pyr
Gender	boys		0	0	0	0	0	0
	girls		-0.169（-0.562，0.225）	-0.206（-0.642，0.231）	0.101（-0.149，0.352）	-0.214（-0.686，0.259）	0.177（-0.248，0.601）	-0.257（-0.619，0.106）
Age （years）			**0.097** **（0.033，0.160）**	**0.103** **（0.032，0.174）**	**0.056** **（0.016，0.097）**	**0.085** **（0.009，0.162）**	**0.098** **（0.029，0.167）**	**0.136** **（0.078，0.195）**
BMI （kg/m^2^）	underweight/ normal		0	**0**	0	**0**	0	0
	overweight/obesity		**-0.538 （-1.022，-0.053）**	**-0.566 （-1.104，-0.028）**	-0.080（-0.388，0.229）	-0.441（-1.023，0.142）	-0.221（-0.744，0.302）	0.067（-0.380，0.514）
Maternal education level	less than high school		0	0	0	0	0	0
	high school		**-0.770 （-1.277，-0.262）**	**-1.126 （-1.690，-0.562）**	-0.313（-0.636，0.010）	**-1.265 （-1.876，-0.655）**	**-0.691 （-1.240，-0.143）**	0.052（-0.416，0.520）
	college or above		**-0.778 （-1.303，-0.252）**	**-1.161 （-1.745，-0.578）**	-0.202（-0.536，0.133）	**-1.361 （-1.993，-0.729）**	**-1.052 （-1.620，-0.484）**	-0.037（-0.522，0.447）
Family income （RMB，Yuan/month）	<3000		0	0	0	0	0	0
	3000-5000		0.045（-0.501，0.591）	0.157（-0.450，0.763）	0.235（-0.113，0.582）	-0.090（-0.747，0.567）	0.379（-0.211，0.969）	0.278（-0.226，0.782）
	5000-8000		-0.284（-0.895，0.326）	0.053（-0.625，0.731）	0.191（-0.198，0.580）	0.092（-0.643，0.826）	0.154（-0.505，0.814）	0.315（-0.249，0.878）
	>8000		-0.420（-1.110，0.269）	0.424（-0.341，1.190）	0.183（-0.256，0.621）	0.675（-0.154，1.504）	0.176（-0.568，0.921）	0.192（-0.444，0.828）
Physical activity （times/week）	≤1		0	0	0	0	0	0
	2–6		0.162（-0.286，0.609）	0.057（-0.440，0.554）	0.027（-0.258，0.312）	-0.038（-0.576，0.500）	0.035（-0.448，0.519）	-0.203（-0.616，0.210）
	≥7		0.277（-0.405，0.958）	0.050（-0.707，0.807）	-0.084（-0.518，0.350）	-0.549（-1.368，0.270）	0.415（-0.321，1.151）	0.247（-0.382，0.875）
Passive smoking	no		0	0	0	0	0	0
	yes		0.129（-0.344，0.603）	0.278（-0.248，0.804）	0.260（-0.042，0.561）	0.398（-0.171，0.968）	0.171（-0.340，0.683）	-0.112（-0.549，0.325）
Diet	red meat （times/week）		-0.023（-0.094，0.047）	-0.075（-0.154，0.003）	-0.018（-0.062，0.027）	-0.081（-0.166，0.004）	-0.044（-0.120，0.032）	-0.054（-0.119，0.011）
	white meat （times/week）		0.048（-0.074，0.170）	0.094（-0.042，0.230）	0.054（-0.024，0.132）	0.103（-0.044，0.250）	0.094（-0.038，0.226）	0.074（-0.039，0.187）
	seafood （times/week）		-0.125（-0.323，0.074）	-0.098（-0.318，0.122）	0.050（-0.076，0.176）	-0.041（-0.279，0.198）	0.032（-0.182，0.246）	0.035（-0.148，0.218）
	vegetable （times/week）		0.053（-0.016，0.122）	**0.088** **（0.012，0.165）**	0.007（-0.036，0.051）	0.028（-0.055，0.111）	0.012（-0.063，0.086）	0.035（-0.028，0.099）
	egg （times/week）		-0.023（-0.127，0.081）	0.011（-0.104，0.127）	0.008（-0.058，0.074）	0.022（-0.103，0.147）	0.012（-0.100，0.125）	-0.041（-0.136，0.055）
	fruit （times/week）		0.024（-0.065，0.112）	-0.002（-0.100，0.096）	-0.004（-0.061，0.052）	0.087（-0.019，0.193）	0.024（-0.071，0.120）	-0.015（-0.096，0.066）
	milk （times/week）		**-0.072 （-0.145，-0.000）**	**-0.087 （-0.167，-0.007）**	0.000（-0.046，0.046）	-0.053（-0.140，0.034）	**-0.095（-0.173，-0.017）**	-0.059（-0.125，0.008）
	bean （times/week）		0.017（-0.081，0.115）	0.036（-0.073，0.146）	0.043（-0.020，0.105）	0.006（-0.112，0.124）	0.074（-0.032，0.180）	0.010（-0.080，0.101）
Water	tap water		0	0	0	0	0	0
	water cooler jug		0.222（-0.269，0.713）	0.172（-0.374，0.717）	**0.462** **（0.149，0.774）**	0.462（-0.128，1.053）	**0.898** **（0.367，1.428）**	0.351（-0.103，0.804）
	purified water		-0.207（-0.802，0.387）	-0.023（-0.684，0.637）	-0.024（-0.403，0.354）	0.023（-0.692，0.738）	0.067（-0.576，0.710）	-0.021 （-0.570，0.528）
	other		-0.064（-1.281，1.152）	0.617（-0.735，1.968）	0.439（-0.336，1.213）	0.270（-1.193，1.733）	0.299（-1.016，1.613）	-0.223（-1.345，0.900）

Values in bold indicated statistical significance （*P*<0.05）.

### 2.4 健康风险评估

#### 2.4.1 非致癌健康风险评估

由于当前的药代动力学模型中，仅芘的相关参数较为完整，因此本研究以芘作为代表性化合物进行非致癌健康风险评估。具体参数参考了Haddad等^［36］^和Viau等^［37］^的研究成果，其中芘的消除速率常数*b*为0.068，吸收率*A*为0.90，吸收剂量分布在血液中的比例为0.052。根据美国环保署（USEPA）公布的数据，芘的参考剂量RfD为30 μg/（kg bw·day）。基于血清中芘的浓度，依据公式（4）和（5）估算其TEDI和HQ。结果显示，芘的TEDI范围为0~0.302 μg/（kg bw·day），中位数为0.004 μg/（kg bw·day）。HQ值均小于1，表明本研究人群血清中芘的暴露水平尚不足以构成明显的非致癌健康风险（见表3）。

**表3:** Pyr的每日总摄入量和HQ

Indicator	P25	P50	P75	P95	Range
TEDI （μg/（kg bw·day））	0.000	0.004	0.034	0.096	0.000-0.302
HQ	0.000	0.000	0.001	0.003	0.000-0.010

#### 2.4.2 致癌健康风险评估

基于BaPeq浓度的致癌风险评估结果显示，蒽和菲具有最高的BaPeq浓度，是儿童及青少年PAHs暴露中致癌风险的主要贡献单体（见表4）。尽管蒽的血清浓度相对较低，但由于其TEF值较高，因此对总体致癌风险的贡献最大，其中位BaPeq浓度为5.39×10^-3^ ng/mL，占总BaPeq的54.6%。菲的中位BaPeq浓度为3.03×10^-3^ ng/mL，占总BaPeq的30.7%，作为血清中浓度最高的PAH，其较大的暴露负荷显著推动了整体致癌风险水平的上升。

**表4:** 各PAH单体的BaPeq浓度

Compound	TEF	BaPeq/（ng/mL）				
		P25	P50	P75	P95	Range
Ant	0.010	1.90×10⁻^3^	5.39×10⁻^3^	1.23×10⁻^2^	4.24×10⁻^2^	3.54×10⁻^5^–1.84×10⁻^1^
Phe	0.001	1.08×10⁻⁴	3.03×10⁻^3^	1.44×10⁻^2^	1.52×10⁻^1^	3.54×10⁻⁶–4.91×10⁻^1^
Flu	0.001	7.07×10⁻⁷	1.06×10⁻^3^	4.41×10⁻^3^	1.85×10⁻^2^	7.07×10⁻⁷–1.01×10⁻^1^
Acp	0.001	7.07×10⁻⁷	3.16×10⁻⁴	1.24×10⁻^3^	7.57×10⁻^3^	7.07×10⁻⁷–5.58×10⁻^2^
Pyr	0.001	7.07×10⁻⁷	3.48×10⁻⁵	3.21×10⁻⁴	9.23×10⁻^4^	7.07×10⁻⁷–2.97×10⁻^3^
AcPy	0.001	7.07×10⁻⁷	4.00×10⁻⁵	4.12×10⁻⁴	3.15×10⁻^3^	7.07×10⁻⁷–1.75×10⁻^2^
ΣPAHs	–	2.01×10⁻^3^	9.87×10⁻^3^	3.31×10⁻^2^	2.25×10⁻^1^	–

TEF： toxic equivalency factor.

## 3 结论

本研究发现，我国西部地区儿童及青少年广泛暴露于PAHs，其中蒽的检出率最高，菲的血清浓度最高。年龄、BMI、母亲教育水平、饮食习惯和饮用水类型是影响儿童及青少年血清中PAHs浓度的重要因素。健康风险评估显示，以芘为代表化合物估算的非致癌风险总体可忽略；致癌风险评估则提示蒽和菲是主要的致癌风险贡献物，其长期低剂量暴露的致癌风险不容忽视。然而，本研究非致癌风险仅基于芘，未涵盖其他PAHs，可能低估实际风险水平。此外，BaPeq方法评估的是致癌潜力而非真实风险概率，存在一定局限性。建议后续研究加强儿童及青少年PAHs内暴露全面监测，完善相关暴露风险评估参数，拓展健康风险评估范围，为精准干预和公共卫生政策制定提供科学依据。
